# Silver polyvinyl pyrrolidone nanoparticles exhibit a capsular polysaccharide influenced bactericidal effect against *Streptococcus pneumoniae*

**DOI:** 10.3389/fmicb.2014.00665

**Published:** 2014-12-03

**Authors:** Ronda K. Bibbs, Rhonda D. Harris, Veolanda A. Peoples, Cleon Barnett, Shree R. Singh, Vida A. Dennis, Mamie T. Coats

**Affiliations:** ^1^Center for NanoBiotechnology Research, Alabama State UniversityMontgomery, AL, USA; ^2^Department of Physical Sciences, Alabama State UniversityMontgomery, AL, USA; ^3^Department of Biological Sciences, Alabama State UniversityMontgomery, AL, USA

**Keywords:** AgPVP, pneumococcus, nanoparticle, serotype, antimicrobial, titanium dioxide

## Abstract

*Streptococcus pneumoniae* remains a leading cause of morbidity and mortality worldwide. The highly adaptive nature of *S. pneumoniae* exemplifies the need for next generation antimicrobials designed to avoid high level resistance. Metal based nanomaterials fit this criterion. Our study examined the antimicrobial activity of gold nanospheres, silver coated polyvinyl pyrrolidone (AgPVP), and titanium dioxide (TiO_2_) against various serotypes of *S. pneumoniae*. Twenty nanometer spherical AgPVP demonstrated the highest level of killing among the tested materials. AgPVP (0.6 mg/mL) was able to kill pneumococcal serotypes 2, 3, 4, and 19F within 4 h of exposure. Detailed analysis of cultures during exposure to AgPVP showed that both the metal ions and the solid nanoparticles participate in the killing of the pneumococcus. The bactericidal effect of AgPVP was lessened in the absence of the pneumococcal capsular polysaccharide. Capsule negative strains, JD908 and RX1, were only susceptible to AgPVP at concentrations at least 33% higher than their respective capsule expressing counterparts. These findings suggest that mechanisms of killing used by nanomaterials are not serotype dependent and that the capsular polysaccharide participates in the inhibition. In the near future these mechanisms will be examined as targets for novel antimicrobials.

## INTRODUCTION

*Streptococcus pneumoniae* (pneumococcus) is an encapsulated, Gram-positive, facultative anaerobic bacterium which is a common commensal in the nasopharynx of healthy adults and children (Zemlickova et al., 2006; Calix et al., 2012; Marks et al., 2013). Asymptomatic carriage of *S. pneumoniae* can lead to invasive and non-invasive infections (Morona et al., 2004; Abdelnour et al., 2009; Ghasemi et al., 2009). Symptomatic disease will occur when the bacterium migrates to normally sterile organs such as the lungs, spinal cord, and middle ear (Murkerji et al., 2012). Such infections have been associated with high morbidity and mortality in young children under 5 years of age, the elderly, and immunocompromised especially in developing countries (Ghasemi et al., 2009; Mehr and Wood, 2012). Annually, in the United States, there are an estimated 500,000 cases of pneumonia, 50,000 cases of bacteremia, 3,000–6,000 cases of meningitis, 7,000,000 cases of otitis media with the major cause being pneumococcus (Temime et al., 2008; Daka et al., 2011; CDC, 2013). It has also been estimated by the World Health Organization that 2 million children, under the age of five, die each year from complications associated with pneumococcal pneumonia including endobronchial obstruction, empyema, and pericarditis (Bryce et al., 2005).

The highly recombinant nature of *S. pneumoniae* in combination with naturally occurring antibiotic resistance and the overuse of antibiotics are all likely to contribute to the organism’s increasing levels of antibiotic insensitivity. While β-lactams remain the preferred treatment for pneumococcal infection, their usefulness is limited due to increasing resistance to several classes of antimicrobials. The morbidity and mortality associated with pneumococcal infections, as well as the ever declining level of antibiotic susceptibility and limited usefulness of prophylaxis, point to the need for novel antimicrobials.

Nanomaterials have shown some usefulness in killing pathogenic bacteria. Some nanoparticles (NPs), typically on the 0.2–100 nm scale, (El-Nour et al., 2010; Beer et al., 2012; Sun et al., 2013) are finding increasing application as antimicrobials and additives in food packaging, industrial and consumer products (Ravishankar and Jamuna, 2011; Sahayaraj and Rajesh, 2011; Ivask et al., 2012). Metal NPs have been of great interest due to their physiochemical properties (e.g., electromagnetic, optic and molecular recognition, and catalytic) that differ from bulk materials of micro-size (Kaittanis et al., 2010; Sadeghi et al., 2010; Ahmad et al., 2013). Higher activity of nanoscale materials is a result of their surface-to-volume ratios. As the material becomes smaller, the percentage of active molecules/atoms present at the surface increases compared to larger bulk material.

The advantages of using metallic NPs are as follows: (1) core of the material is inert, (2) exploitation of both chemical and physical properties enables them to carry and release various therapeutic agents, (3) synthesis is relatively simple and (4) mechanism of action allows versatility. Once the metal is ionized, it becomes very reactive as it interacts with thiol group of respiratory enzymes that are located in the bacterial cell wall or cell membrane (Ghosh et al., 2008). When the NPs enter the cell, they will migrate to the center of the bacteria. The bacteria conglomerate in an effort to protect its DNA from the metal ions. This event will eventually cause the DNA to condense leading to the retardation of the respiratory chain, cell division, and finally cell death (Rai et al., 2009; Sadeghi et al., 2010; Arshi et al., 2011). Microbes would need to acquire a range of mutations to gain resistance against the metals effects. This makes NPs good candidates as antimicrobial agents.

The goals of the current study were to examine the antimicrobial effect of metallic NPs against clinically relevant serotypes of *S. pneumoniae* and to determine if the capsular polysaccharide plays a role in the inhibition. Each of these findings is novel to *S. pneumoniae* research.

## MATERIALS AND METHODS

### NANOPARTICLE AND SYNTHESIS

Silver polyvinyl pyrrolidone (AgPVP) coated NPs (20 nm) and titanium dioxide (Anatase; 15 nm) were purchased from Nanostructured and Amorphous Materials, Inc. (Houston, TX, USA). Gold nanospheres were synthesized from hydrogen tetrachloroaurate (III), HAuCl_4_, (Sigma Aldrich; Kimling et al., 2006). Gold hydrochlorate (HAuCl_4_) was dissolved in deionized water and heated to 100∘C with vigorous stirring. Heated sodium citrate was added and allowed to stir for 30 min. The color of the solution gradually changed from faint yellow to gray then deep purple before becoming wine red. The process produced spherical 20 nm particles. All NPs were examined using transmission electron microscopy (TEM).

### BACTERIA STRAINS AND CULTURING

RX1 (Morona et al., 2004; Marks et al., 2012; Murkerji et al., 2012) was a gift from David E. Briles at the University of Alabama at Birmingham, Birmingham, AL, USA. The capsular polysaccharide deficient *S. pneumoniae* strain JD908 (Magee and Yother, 2001; Marks et al., 2012) was provided by Janet Yother at the University of Alabama at Birmingham, Birmingham Alabama. JD908 was grown in the presence of erythromycin (0.3 μg/mL). All *S. pneumoniae* strains used in the study are listed in **Table 1**. Strains were grown in Todd Hewitt broth supplemented with 0.5% yeast extract (THY). Bacteria were quantitated and stored at -80∘C until use.

**Table 1:** *Streptococcus pneumoniae* strains included in the study.

Name	Serotype	Background strain	Penicillin	Erythromycin	Reference
D39	2	NA	Sensitive	Sensitive	Magee and Yother (2001), Sanders et al. (2011), Murkerji et al. (2012)
WU2	3	NA	Sensitive	Sensitive	Magee and Yother (2001), Li et al. (2009), Ren et al. (2012)
TIGR4	4	NA	Sensitive	Sensitive	Weinberger et al. (2009), Melin et al. (2010), Murkerji et al. (2012)
EF3030	19F	NA	Sensitive	Sensitive	Johnston et al. (2006), Coats et al. (2011)
RX1	2	D39	Sensitive	Sensitive	Morona et al. (2004), Marks et al. (2012), Murkerji et al. (2012)
JD908	3	WU2	Sensitive	R (0.03 μg/mL)	Magee and Yother (2001), Marks et al. (2012)

### DETERMINATION OF ANTIMICROBIAL ACTIVITY IN LIQUID CULTURE

In a variation of the microdilution technique, *S. pneumoniae* cultures were diluted to 1.0 × 10^3^ CFU/mL in THY and grown in 4 mL cultures in the presence of various concentrations of metallic NPs at 37∘C for 5 h to determine the minimum bactericidal concentration (MBC) of the NPs (Gnanadhas et al., 2013; Yadav et al., 2013). The 5 h incubation was chosen because our preliminary data showed that in cultures where complete killing was seen, cell death occurred prior to the 5 h time point. Following incubation, serial dilutions of the bacteria were plated on tryptic soy agar supplemented with 5% sheep blood. Following overnight incubation at 37∘C, the bacteria were quantitated.

### QUANTITATION OF METAL ION RELEASE FROM NANOPARTICLES

Titanium dioxide (95 mg/mL), AgPVP (0.6 mg/mL), and activity of gold nanospheres (AuNP; 1 mg/mL) were incubated individually in THY media for 5 h at 37∘C. Ag and Au ions released into the media were measured using laser induced breakdown spectroscopy (LIBS). The measurement settings were a modification of Barnett et al. (2011). The excitation wavelength used was 266 nm and the laser energy was adjusted to 20 mJ. The spectrometer and detector were changed to the Andor Shamrock 303i – iStar – DH320T-25F-03 with gating at 2400 l/mm. Data was collected approximately 1.5 μs after the plasma started and the signal was integrated for 3.0 μs. Gold was monitored using Au I at 267.60 nm and while silver was monitored using Ag I at 328.07 and 338.29 nm.

Ti was measured using a standard curve and measuring the optical density at 360 nm.

### CHARACTERIZATION OF *S. pneumoniae*/AgPVP INTERACTIONS

*Streptococcus pneumoniae* D39 (1.0 × 10^3^ CFU/mL) was grown in THY in the presence of AgPVP (0.6 mg/mL) NPs and incubated at 37∘C for 5 h. At various time points, cultures were pelleted by centrifugation for 15 min at 2012 × *g*. After centrifugation, the supernatant was removed and the pellet was washed using phosphate buffer saline (1XPBS) then pelleted. Bacteria were fixed using 2.5% Glutaraldehyde, 1% osmium tetroxide, and 1XPBS. Images were captured via Scanning electron microscopy (ZEISS EVO 50VP).

### STATISTICS

Differences between *S. pneumoniae* (WT) strains were compared with their capsular negative mutants using a two sample, independent Student *t*-test. A *P* value of <0.05 was considered significant.

## RESULTS

### ANALYSIS OF NANOMATERIALS

**FIGURE 1:**
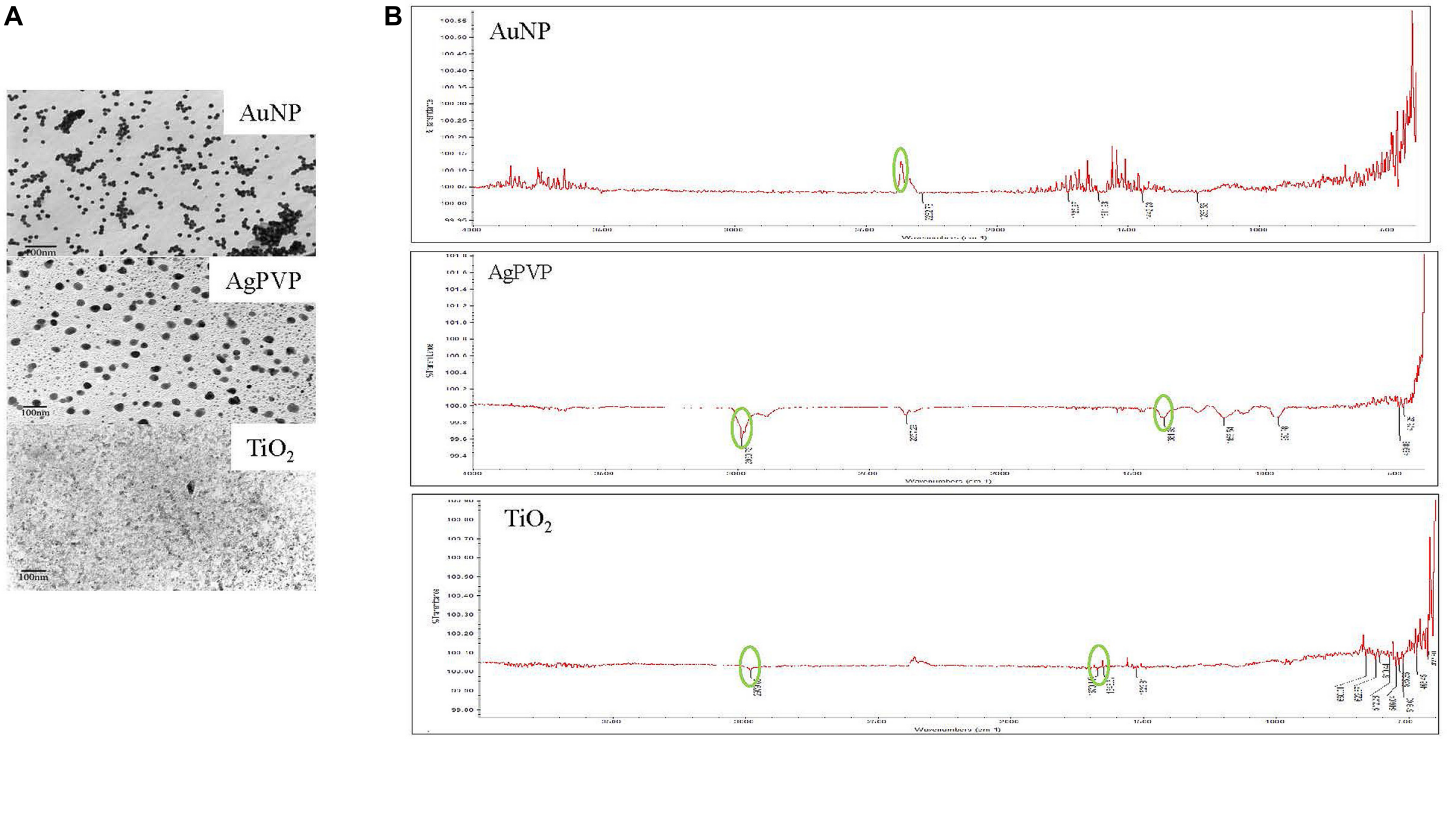
**Characterization of nanoparticles. (A)** TEM Images of AuNP, AgPVP, and TiO_2_ nanoparticles. Nanomaterials were deposited onto a copper grid for imaging. **(B)** FT-IR spectra of nanoparticles. Most relevant peaks are circled.

Transmission electron microscopy showed the AuNP, AgPVP, and TiO_2_ to be 20 nm, 20 nm, and 15 nm respectively, with uniform particle distribution (**Figure 1A**). All particles were spherical. The FT-IR spectra for each NP correlate with the fingerprint expected for the respective particle (**Figure 1B**) (Huang et al., 2010; Tiwari et al., 2011; Devika et al., 2012).

### MINIMUM BACTERICIDAL CONCENTRATIONS

**FIGURE 2:**
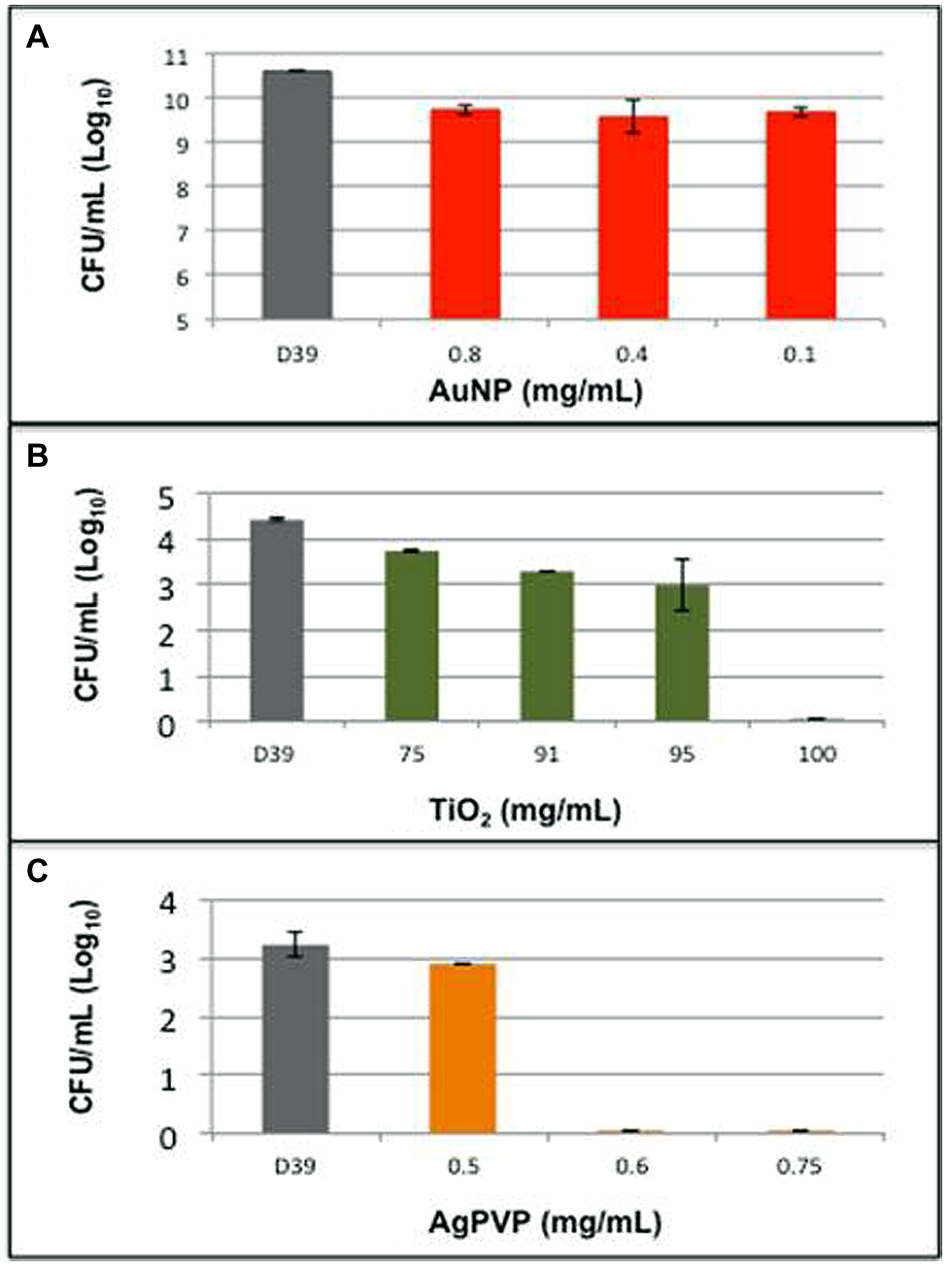
**Metallic nanoparticles have varying effects on pneumococcal growth.** Pneumococcal strain D39 was grown in liquid media in the presence of **(A)** gold nanospheres (AuNP), **(B)** titanium dioxide (TiO_2_), and **(C)** silver coated polyvinyl pyrrolidone (AgPVP). Bars represent the Log_10_ of the mean number of surviving organisms following a 5 h growth period. Errors are the SD of grouped data.

*Streptococcus pneumoniae* strain D39 was grown in liquid media in the presence of varying concentrations of AuNP, AgPVP, or TiO_2_. The minimum concentrations of TiO_2_ and AgPVP needed to kill *S. pneumoniae* D39 were determined to be 95 mg/mL and 0.6 mg/mL respectively, (**Figure 2**). AuNP at concentrations up to 0.8 mg/mL were able to cause a maximum decrease of 9% in the culture’s viability. Higher concentrations of AuNP were not examined because the bactericidal effect plateaued at concentrations above 0.4 mg/mL (**Figure 2**). When AgPVP and TiO_2_ were examined for their ability to kill various *S. pneumoniae* strains of varying serotypes, AgPVP gave more consistent effects than TiO_2_ (data not shown). All tests were done in duplicate and repeated twice. The bactericidal action of metallic NPs is largely attributed to the release of metal ions from the particle. The levels of Ag, Au, and Ti in the culture media at the end of the 5 h incubation period were determined to be <2.5 μg/mL, 0.0078 μg/mL and 20 mg/mL respectively.

### NON-SEROTYPE DEPENDENT ANTIBACTERIAL ACTIVITY OF AgPVP

**FIGURE 3:**
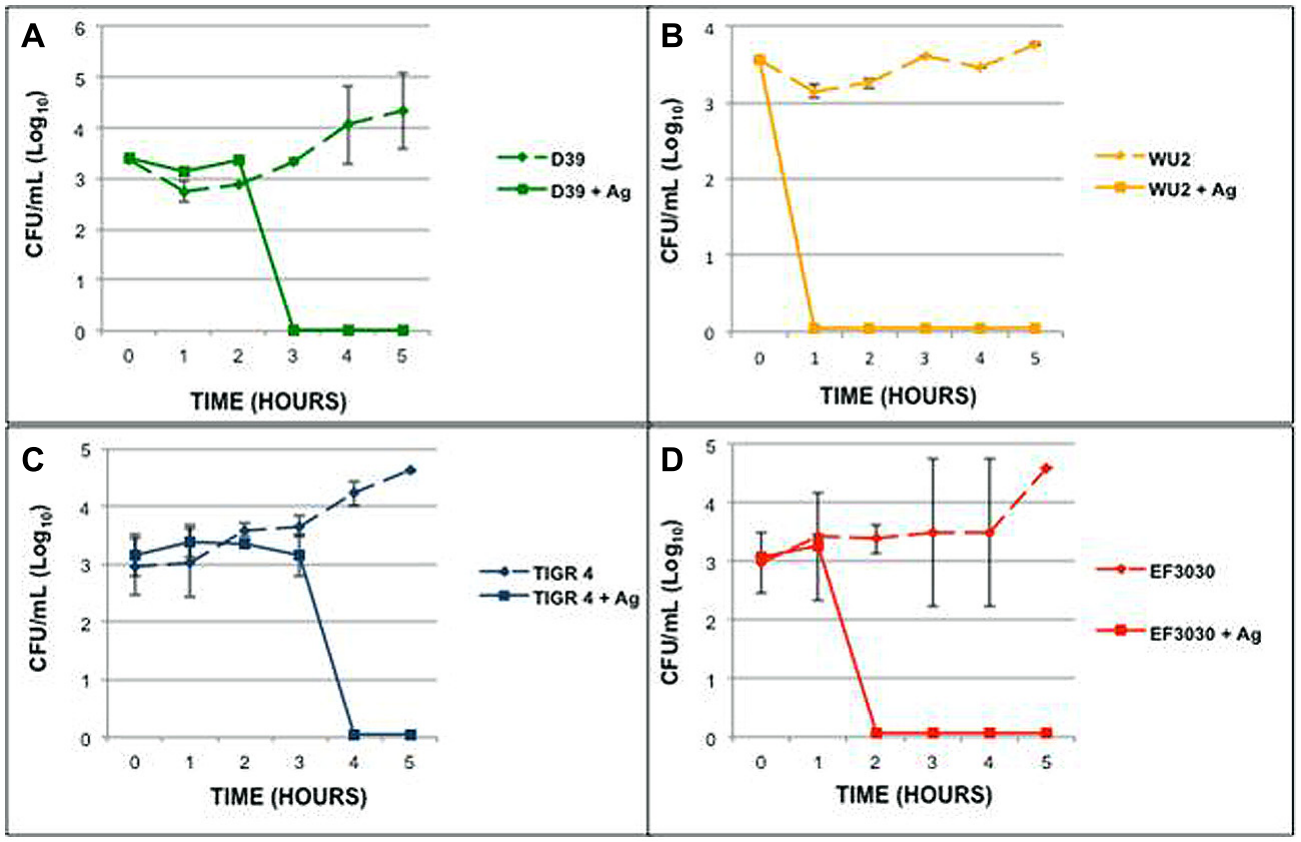
***Streptococcus pneumoniae* strains of different serogroups respond differently in the presence of Ag-PVP.** Strains **(A)** D39 (serotype 2), **(B)** WU_2_ (serotype 3), **(C)** TIGR4 (serotype 4), and **(D)** EF3030 (serotype 19F) were grown in the presence of Ag-PVP (0.6 mg/mL). Samples were tested in duplicate and repeated twice. Symbols represent the average viable CFU (Log_10_) and the SD of grouped data.

The 93 serotypes of *S. pneumoniae* are not equal in their ability to cause disease or their behavior *in vitro*. In order to determine the effect of serotype on the antimicrobial effect of metallic nanomaterials, four pathogenic *S. pneumoniae* serotypes were tested for the ability to survive in the previously determined MBC of AgPVP. *S. pneumoniae* isolates D39 (serotype 2), WU2(serotype 3), TIGR4 (serotype 4), and EF3030 (serotype 19F) were grown in the presence of AgPVP (0.6 mg/mL; **Figure 3**). While all the tested isolates were susceptible to AgPVP at the test concentration, the time required to completely kill the cultures varied. Also, while the time to death varied between the individual strains none survived more than 4 h. Due to the variation in survival times for the individual strains representing the various serotypes, additional strains within the serogroups were tested for survival in the presence of 0.6 mg/mL AgPVP. The length of time individual strains survived varied among strains of the same serotype (data not shown). These findings together suggest that inhibition is not dependent on the serotype.

### CAPSULAR POLYSACCHARIDE INFLUENCED KILLING OF *S. pneumoniae*

**FIGURE 4:**
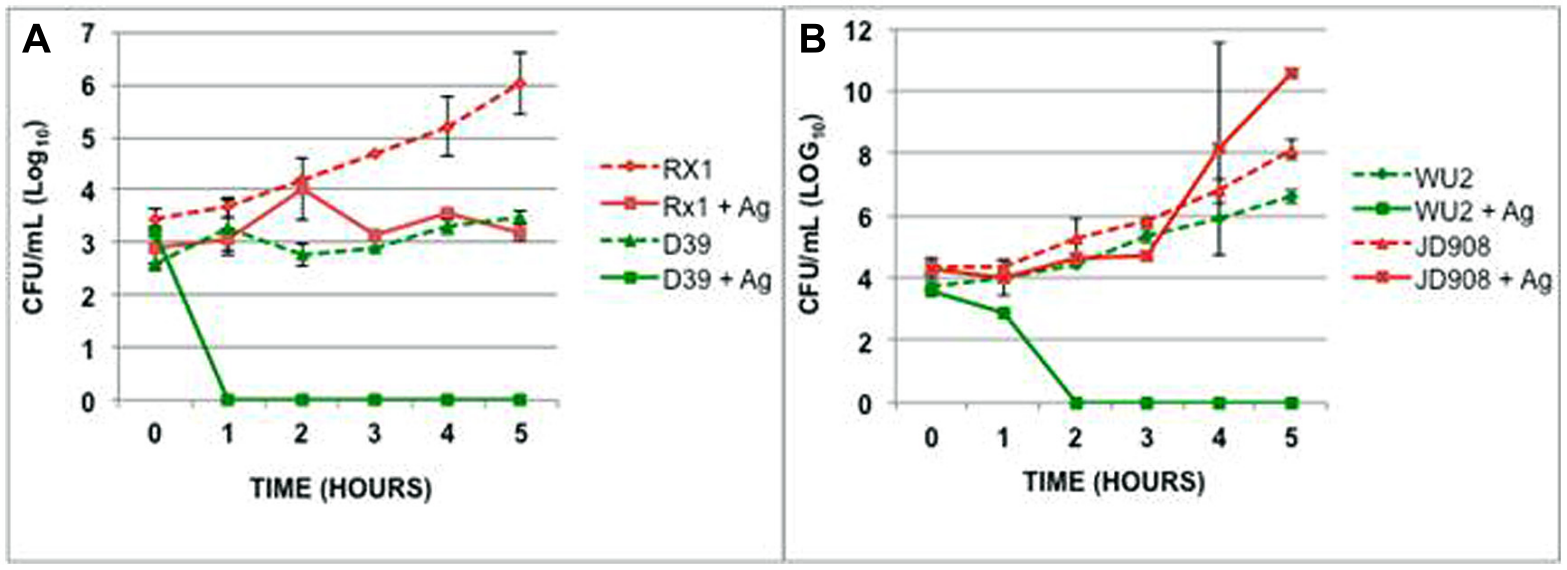
**Capsular polysaccharide contributes to *S. pneumoniae*’s sensitivity to AgPVP. (A)** D39, serotype 2 and **(B)** WU2, serotype 3, along with their capsule polysaccharide negative mutants were grown in the presence of 0.6 mg/mL AgPVP nanoparticles for 5 h. Rx1 survived in significantly higher numbers than D39 (*P* = 0.0005) as did JD908 when compared to WU2 (*P* = 0.003).

The variability in time to death during exposure to AgPVP for the individual serotypes suggests that the capsular polysaccharide may play a role in the interaction between bacteria and NPs. In order to examine the effect of the pneumococcal capsule on the antimicrobial activity of metallic NPs, *S. pneumoniae* D39, WU2 and their capsular polysaccharide negative mutants, Rx1 and JD908, respectively, were grown for 5 h in the presence of AgPVP (**Figure 4**). Rx1 and JD908 were found to survive significantly longer and in higher numbers than their respective wild-type counterparts (*P* = 0.0005 and *P* = 0.003, respectively). In fact, the mutant strains lacking capsular polysaccharide remained viable in the presence of AgPVP. RX1 maintained the number of viable colonies while JD908 increased in CFU. Increasing the incubation time did not result in death of the mutant strains due to AgPVP (data not shown). AgPVP concentrations of 0.8 mg/mL and higher were able to kill RX1 and JD908 *S. pneumoniae*. The growth patterns and responses to AgPVP suggest that the capsular polysaccharide participates in the mechanism used by AgPVP to kill *S. pneumoniae*.

### ULTRASTRUCTURAL EFFECT OF AgPVP

**FIGURE 5:**
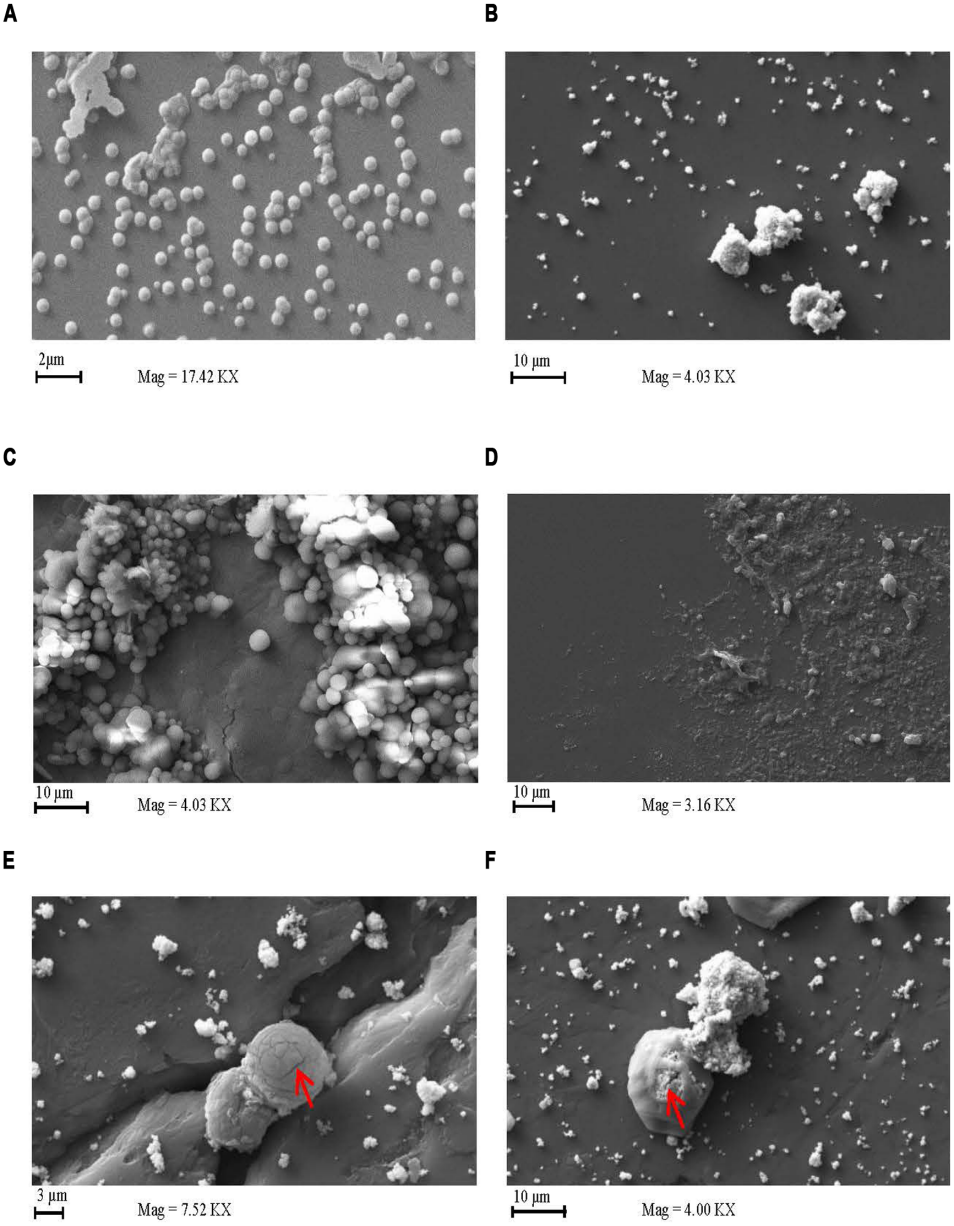
**Silver coated polyvinyl pyrrolidone has a lytic effect on *S. pneumoniae.*** Scanning electron microscopy images of *S. pneumoniae* grown in the presence and absence of AgPVP. *S. pneumoniae* D39 was exposed to AgPVP for 0 **(A)** and 5 h **(C–F)**. Cultures exposed to AgPVP were compared to unexposed samples **(C)**. AgPVP particles alone imaged as well **(B)**. Samples were deposited onto a glass slide mounted on a stud for imaging.

Scanning electron microscopy of *S. pneumoniae* D39 grown in the presence of AgPVP demonstrated the killing of the bacterial cells in the presence of the nanomaterial (**Figure 5D-F**). AgPVP alone is shown in **Figure 5B**. Images were taken at 0 h (**Figure 5A**) and 5 h incubation (**Figure 5C-F**). The cell debris remaining following exposure of the bacteria to AgPVP is shown in **Figure 5D**. **Figure 5C** shows bacteria grown in the absence of AgPVP. The absence of cell debris in the **Figure 5C** sample indicates that the debris seen in **Figure 5D** is not attributable to autolysis of the bacteria. The damage and breakdown of the bacterial capsular polysaccharide and cell wall can be seen in **Figure 5E** and is indicated by the arrow. Also, while much of the damage done by metallic NPs is thought to be due to the metal ions, the NPs themselves do participate in the inhibition of the bacteria as is seen in **Figure 5F** which shows AgPVP inside the bacteria (indicated by arrow). As the particles accumulate in the bacteria the bacteria increase in size and eventually lyse.

## DISCUSSION

For decades, metal ions and metal based compounds have been used in various capacities due to their antimicrobial properties against different pathogenic microorganisms (Zhou et al., 2012; Ahmad et al., 2013; Lima et al., 2013). The present study is the first detailed examination of the potential use of metallic NPs against clinically relevant serotypes of the formidable human pathogen, *S. pneumoniae*. Due to their varying modes of action, Au, Ag, and TiO_2_ NPs were examined for antimicrobial effects against *S. pneumoniae*. AgNP showed the most reproducible inhibition. This is likely due to multifaceted damage caused AgNPs including condensation of DNA, cell membrane separation from the cell wall, and cell wall damage (Morones et al., 2005; Seil and Webster, 2012; You et al., 2012). The levels of AgNPs needed to inhibit pneumococcal growth are higher than those reported for other microorganisms (Pelgrift and Friedman, 2013; Losasso et al., 2014). This is likely due to the unique physical characteristics of the bacterium. The lack of inhibition by AuNPs is likely because their antimicrobial effect is due to the targeting of sulfur-containing proteins present in the membrane or inside the cells but *S. pneumoniae* does not contain many such proteins. Also, a much smaller percentage of Au ions were released into the media. TiO_2_ is more limited in its activity due to the need for activation by UV light or the availability of doped catalysts (Kuhn et al., 2003; Pelgrift and Friedman, 2013).

The role of the capsular polysaccharide in protecting the pneumococcus is well documented (Magee and Yother, 2001; Melin et al., 2010; Sanders et al., 2011). The capsular is also a major contributor for the organism’s pathogenicity and is the basis for the 93 known antigenically distinct serotype classifications (Hyams et al., 2010; Hathaway et al., 2012; Wyres et al., 2012). Disease serotypes 2, 3, 4, and 19F were all found to be susceptible to the antimicrobial effects of AgPVP. These serotypes are particularly relevant because isolates of each serotype have been shown to be virulent in animal models of infection as well as human disease (Coats et al., 2011; Hotomi et al., 2013; Okade et al., 2014). This finding supports the likelihood that other disease causing serotypes will be susceptible as well. Having broad range applicability to pneumococcal infection makes NPs far more attractive in the war against pneumococcal infection.

During infection, the capsule protects *S. pneumoniae* from opsonophagocytosis. This protective role of the capsule can appear counterintuitive when considered alongside our data showing that *S. pneumoniae* strains completely lacking a capsular polysaccharide were less susceptible to the effects of metallic nanomaterials. In reality, this finding suggests a yet undefined role of the capsule in the presence of metallic ions and possible nanomaterials in general. One possible mechanism is the capsule aiding in the accumulation of metal ions near the cell wall where they can more easily access the cell membrane. A second possibility involves the capsule participating in the movement of metal ions or whole NPs into the bacterial cells.

Additionally, while the capsule polysaccharide appears to participate in killing of the pneumococcus by nanomaterials this does not undermine the use of these nanomaterials in the possible treatment of pneumococcal disease. This is because the capsule polysaccharide is essential for infection by the pneumococcus (Hammerschmidt et al., 2005; Weinberger et al., 2009; Hyams et al., 2010). Thus if the bacteria respond to the presence of an antimicrobial by decreasing its’ capsular polysaccharide, the bacteria would be rapidly cleared by the immune system.

Following exposure to AgPVP, there was a decrease in the viable number of *S. pneumoniae*. This strongly suggests that the bacteria were killed by the NP. Cell death is further supported by the cell lysis that was visible when the culture was examined using microscopy. The breakdown of the cell capsular polysaccharide and cell wall are irreversible and conclusively shows that the organism was not in a viable but non-cultureable state.

The behavior of organisms *S. pneumoniae* in the presence of various nanomaterials provides valuable insight into the possibility of novel antimicrobials to treat pneumococcal diseases. While AgPVP is not likely to be used to treat *S. pneumoniae* infection due to its documented toxicity of Ag and AgNPs (Ahamed et al., 2010; Asharani et al., 2011; Hamilton et al., 2014) the identified vulnerability of different pathways used by AgPVP to inhibit growth can give new areas on which to focus design of new drugs.

## CONCLUSION

The results obtained show spherical AgPVP to be preferential to AuNP and TiO_2_ for the killing of *S. pneumoniae*. The inhibition occurred within 4 h of exposure to the nanomaterial, was bactericidal in nature and involves the presence of the capsular polysaccharide.

The susceptibility of several disease relevant pneumococcal serotypes to inhibition by AgPVP is particularly encouraging due to the variability of microorganism. The precise role of the capsular polysaccharide in the killing of *S. pneumoniae* needs to be examined. This will require the examination of organisms expressing various levels of the capsule as well as organisms whose serotype has not been identified.

## Conflict of Interest Statement

The authors declare that the research was conducted in the absence of any commercial or financial relationships that could be construed as a potential conflict of interest.
